# QD-Based FRET Probes at a Glance

**DOI:** 10.3390/s150613028

**Published:** 2015-06-04

**Authors:** Armen Shamirian, Aashima Ghai, Preston T. Snee

**Affiliations:** Department of Chemistry, University of Illinois at Chicago, 845 W. Taylor St., Chicago, IL 60607-7061, USA; E-Mails: ashami2@uic.edu (A.S.); aashimag7@gmail.com (A.G.)

**Keywords:** semiconductor quantum dots, Förster resonance energy transfer, optical sensors, photoluminescence

## Abstract

The unique optoelectronic properties of quantum dots (QDs) give them significant advantages over traditional organic dyes, not only as fluorescent labels for bioimaging, but also as emissive sensing probes. QD sensors that function via manipulation of fluorescent resonance energy transfer (FRET) are of special interest due to the multiple response mechanisms that may be utilized, which in turn imparts enhanced flexibility in their design. They may also function as ratiometric, or “color-changing” probes. In this review, we describe the fundamentals of FRET and provide examples of QD-FRET sensors as grouped by their response mechanisms such as link cleavage and structural rearrangement. An overview of early works, recent advances, and various models of QD-FRET sensors for the measurement of pH and oxygen, as well as the presence of metal ions and proteins such as enzymes, are also provided.

## 1. Introduction

Among various bioimaging/biosensing techniques, fluorescence-based methods are generally preferred over absorption [1,2], especially with the ability to detect single photons with avalanche photodiodes and intensified camera systems [3]. One must employ an emissive chromophore such as an organic dye or fluorescent protein; however, they generally suffer from a lack of photochemical stability and may require sophisticated tunable excitation sources. The effect is to reduce researchers’ ability to perform studies that require multiplex detection over long timescales [4]. Quantum dots (QDs) are semiconductor nanocrystals with unique electro-optical properties due to quantum confinement effects [5]. Specifically, they have broad absorption spectra with large extinction coefficients, narrow and tunable photoluminescence (PL), high emission quantum yields, improved resistance against photobleaching, and long fluorescence lifetimes. As a result, QDs are exceptional bioimaging and biosensing tools [6,7,8,9,10]. As a comparison of QDs and organic dyes as fluorescent labels has been discussed in a recent review [11], we only summarize the main differences here.

Although the aqueous synthesis of QDs results in instantly water soluble materials, the crystalline quality, quantum yield, and the emission bandwidth of the dots are sub-optimal. On the other hand, the synthesis of hydrophobic QDs in organic solvents imparts a higher quantum yield, enhanced monodispersity, and a narrower emission bandwidth [12]. As such, this review focuses on hydrophobic as-prepared dots. One of the fundamental aspects of manufacturing QD-based sensors is water solubilizing the organic capped QDs and functionalizing them for conjugation to biologicals or organics such as dyes. Furthermore, surface passivation via an inorganic shell that covers the cores can reduce the surface defects significantly and improve the QDs photoluminescence properties [13,14,15]. The core/shell structure also improves the stability of the QDs and reduces cytotoxicity by preventing the leaching of toxic elements, generally cadmium, from the cores.

Transferring organic capped QDs into an aqueous solution and functionalizing them was a significant challenge for many years [16]. Several water solubilizing methods have been developed over the past decades, with the majority falling under the categories of micelle encapsulation [17,18,19,20], ligand exchange [21,22,23,24,25], and silanization [26,27,28]. The water soluble capping layer on the surface of the QDs usually possesses reactive groups that can be used for conjugation of target molecules using variety of available chemistries. Historically and generally still true, carboxylic acid groups are the part of the transfer agent that renders as-prepared hydrophobic QDs soluble in water at a physiological pH of 7.4 and engenders biological functionalization with proteins via amide bond formation. Generally commercially-available carbodiimide crosslinking reagents such as 1-ethyl-3-(3-dimethylaminopropyl) carbodiimide (EDC) are used [29]. Coupling efficiencies may be enhanced with the addition of *N*-hydroxysuccinimide (NHS) or sulfo-NHS. However, charge cancellation of anionic QDs with cationic species can render the colloidal dots unstable, causing them to precipitate from water [23,30]. This effect is mollified by either cancelling out the electrostatics of either the dots or the reagents using poly(ethylene glycol), [30,31] which prevents the loss of products and an enhancement of reaction yields. Sulfhydryl coated QDs may be functionalized using a reagent such as sulfosuccinimidyl-4-(*N*-maleimidomethyl)cyclohexane-1-carboxylate (Sulfo-SMCC) which is an amine to thiol cross-linker [32]. Other conjugation approaches based on biotin-streptavidin interactions [33] or self-assembly of polyhistidine tagged molecules on the zinc-rich surface of the QDs [34,35,36] are also frequently reported.

Herein we will address some recent developments of QD-based fluorescent resonance energy transfer (FRET) sensors, including a brief discussion on the basic principles of FRET and an overview of the most commonly used sensing strategies and schemes for the detection of a wide variety of analytes. As the entirety of literature examples on QD-based sensors is very large, we will only highlight just a few examples to limit the scope on motif rather than to create a full list of analytes that have been targeted using the same.

## 2. FRET Principle

Fluorescence resonance energy transfer is a non-radiative transition of energy from an excited dipolar species (the donor) to an acceptor that is in close proximity. FRET is dependent on the distance between the donor and the acceptor, typically in the range of 1–10 nm, as well as the spectral overlap of the donor emission with the acceptor absorption. The FRET efficiency (E) can be defined as:

(1) $$\text{E}=\frac{k_{D-A}}{k_{D-A}+k_{D}}=\frac{{R_{0}}^{6}}{{R_{0}}^{6}+r^{6}}=1-\frac{I_{DA}}{I_{D}}$$

where *k_D-A_* is the donor decay rate in the presence of an acceptor, *k_D_* is the donor decay rate in the absence of an acceptor, *R*_0_ is the donor-acceptor distance at which FRET efficiency is 50% that is dependent on the donor emission overlap with the acceptor absorption, *r* is the actual donor-acceptor distance, *I*_DA_ is the integrated emission intensity of the donor in the presence of an acceptor, and I_D_ is the same without an acceptor. The relationship is fundamentally based on Fermi’s Golden Rule for interacting dipoles, and carries with it all the approximations that the theory is dependent upon. For example, the 6th power of distance scaling behavior is the result of the inverse *r*^3^ dependence of dipole-dipole interactions and the squared coupling term of Fermi’s Golden Rule. Not all types of energy transfer conform to this mechanism and thus have different distance dependencies. Furthermore the donor state cannot be extremely short lived and 1st order time-dependent perturbation theory needs to be applicable, *i.e.*, back energy transfer from acceptor to donor should be minimal. Regardless of the nature of the energy transfer process, the timescale(s) of luminescence rise and decay change when energy transfer is occurring. Obviously the donor lifetime is quenched while the acceptor lifetime is enhanced; this is generally considered the best evidence of FRET. 

Studies have shown that QDs are effective energy donors to organic dye acceptors in a wide range of FRET-based processes [37,38,39] due to their high quantum yields and extinction coefficients, narrow and symmetrical emissions, and photochemical stability. This is somewhat surprising as the large size of nanocrystals would seemingly force the donor-acceptor distance to be so far (>several nm) as to preclude any significant efficiency. The mechanism of the energy transfer process is due to a dipole-dipole interaction [40] and follows the standard *r*^6^-dependent FRET efficiency. Regardless, there are some alterations to the standard model that need to be implemented. For example, it is not feasible to construct a single QD donor-single dye acceptor system. In this case, the FRET efficiency where a single QD donor interacts with *n* acceptors simultaneously and all the acceptors are located at the same distance can be expressed by [41]:

(2) $$E\left(n,\;r\right)=\frac{n{R_{0}}^{6}}{n{R_{0}}^{6}+r^{6}}$$

This is further complicated by the fact that there should be a heterogeneous distribution of the *n* acceptors per QD that follows Poissonian statistics [42], where the probability of a QD having *n* acceptors is:

(3) $$P\left(n,\text{λ}\right)=\frac{{\text{λ}}^{n}e^{-\text{λ}}}{n!}$$

where λ is the average dye:QD ratio. There are a few other issues to note, such as the odd fact that the FRET efficiency calculated from time-resolved data is rarely equivalent to that measured by total donor emission quenching (Equation (1)). This is likely due to the complex nature of QD donor lifetimes which are generally multi-exponential in nature. Last, QDs are not good FRET acceptors from organic dye donors [43]. This was conjectured to result from the longer excitation lifetime of the QD (>10 ns) compared to organic dyes (<10 ns). This is consistent with a breakdown of Förster theory due to the non-applicability of some approximations used in its derivation as discussed above. However, the use of the QDs as acceptors with longer lifetime donors such as lanthanidesis efficient [44,45]. Furthermore, new and interesting variations of energy transfer called bioluminescence- and chemiluminescence- resonance energy transfer have been recently demonstrated. In these systems, biologically emissive proteins conjugated to QDs create “self-lighting” dot systems that require chemical, rather than physical, excitation. 

The use of QDs as donors in FRET systems offers several advantages over conventional fluorophores [46,47,48]. Nanocrystals can be excited at regions far from the acceptor’s absorption spectrum. This minimizes the direct excitation of the acceptor that otherwise would complicate data analysis. The emission spectra of QDs are narrow, tunable, and symmetric allowing for the engineered maximization of the overlap with the acceptor absorption without tailing into the acceptor emission region. Last, QDs can be multifunctionalized to impart both biological targeting and sensing functionalities.

## 3. The Response Mechanisms of QD-Based FRET Sensors: Displacement

### 3.1. Turn-On Sensors 

**Figure 1:**
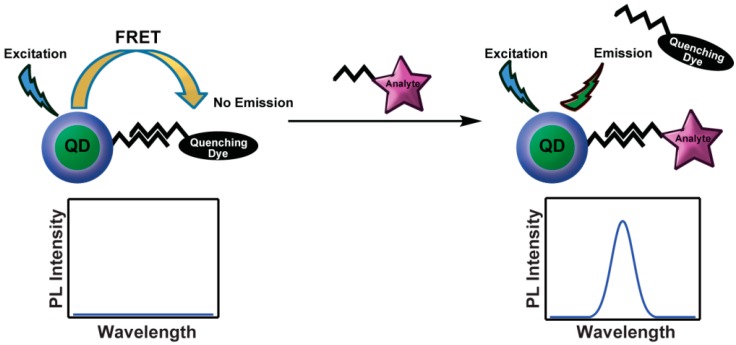
Competitive displacement of a quenching dye by the analyte removes the FRET interaction, resulting in QD emission recovery.

Since the theoretical prediction of energy transfer from QDs to organic dyes [49] and experimental realization [50], numerous examples of the synthesis and study of QD–FRET coupled chromophores have been reported. Generally this work was motivated for sensing, where the design is tailored to the chemical nature of the analyte. The Mattoussi group pioneered the use of QD scaffolds for the development of FRET assays via self-assembly of dye-labelled proteins onto QDs [41]. One of their first reports was based on the competitive binding of a quencher with the target analyte. Initially, QD emission is highly suppressed due to the proximity of a strong quencher that is weakly bound to the protein. Upon addition of the analyte, the competitive displacement of the quenching dye with the target molecule results in a concentration-dependent recovery of the QD emission (Figure 1). This type of reporting results in a so-called “turn-on sensor”. While somewhat difficult to calibrate in a complex system (*i.e.*, *in vivo*), turn on sensing is most useful for the absolute detection of a disease-state biological marker. 

This strategy was first demonstrated by sensing maltose by Medintz *et al.* [51]. In this study, CdSe/ZnS QDs were used as a scaffold for the self-assembly of polyhistidine-terminated maltose binding protein (MBP-His) prebound to β-cyclodextrin that was labeled with a quenching dye. Initially, the quencher rendered QD emission inefficient; however, the displacement of the β-cyclodextrin with maltose resulted in the recovery of the QD PL emission. A similar rationale was used to detect the presence of trinitrotoluene (TNT) [52]. An antibody for TNT that was expressed with an oligohistidine tag was coordinated to CdSe/ZnS QDs in water. This complex was exposed to trinitrobenzene (TNB) labeled with BHQ10 quenching dye which resulted in QD quenching, which was recovered by the addition of TNT samples. This report was significant as it demonstrated the generality of the method through the use of an engineered antibody. 

### 3.2. Ratiometric Sensors, Cleaving the Link

**Figure 2:**
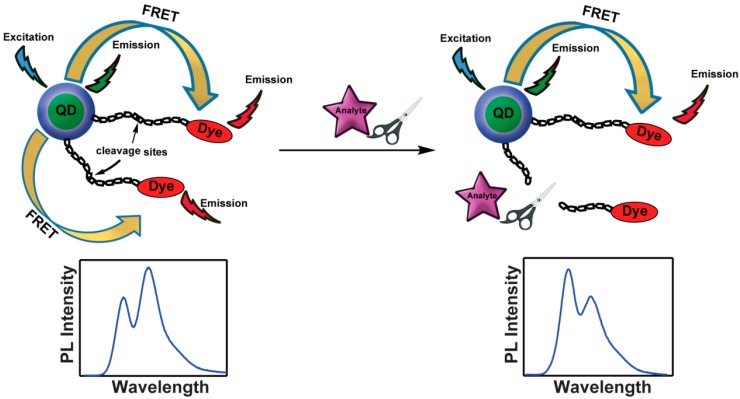
Cleavage of the QD-dye linker by the activity of the analyte causes a disruption of FRET, and a subsequent alteration of the QD-dye emission ratio.

The strategy of donor-acceptor spatial modulation has been used to create a ratiometric response, which is essentially a color change as the reporting mechanism rather than emission intensity modulation. An early demonstration concerned the measurement of proteolytic enzyme activity. Here, a QD is functionalized with a peptide linker that is terminated with an energy accepting fluorescent dye. Due to a finite FRET efficiency, emission can be observed from both the QD donor and dye acceptor. However, the ratio of the intensities of the two chromophores is altered by the addition of an analyte, which cleaves the linker causing the dye to diffuse away from the QD. As a result, nanocrystal emission becomes more dominant (Figure 2). 

Due to their regulatory role on functionality of many proteins involved in major human diseases such as cancer and AIDS, proteases and metalloproteases are the most studied enzymes using this strategy [53,54,55,56,57,58,59,60,61,62,63,64,65,66,67]. Medintz *et al.* employed this strategy for sensing caspase-1, thrombin, chymotrypsin, and collagenase using engineered peptides [68]. Specifically, they synthesized a peptide containing a terminal polyhistidine unit for QD conjugation, then a protease-specific cleavage sequence, and ending with cysteine that was a functional handle for dye conjugation. Addition of the dye functionalized peptides to aqueous dots resulted in enhanced dye emission and QD quenching due to energy transfer. Enzyme addition cleaves the peptides, causing QD-dye displacement and a loss of FRET efficiency that caused a resurgence in the QD emission. A similar motif was constructed by Rao and coworkers [69] for detection of β-lactamase in which an enzyme substrate labeled with carbocyanine dye (Cy5) was immobilized on the QDs through biotin-streptavidin binding. This study was important as the group reported that the length of the linkage and its coverage density on the QD surface affect the rate of enzyme activity. Stevens and coworkers reported a simple detection method for histone acetyltransferase [70]. The group also demonstrated the first multiplex sensing mechanism for simultaneous detection of proteases and kinases using a QD-FRET probe [71].

**Figure 3:**
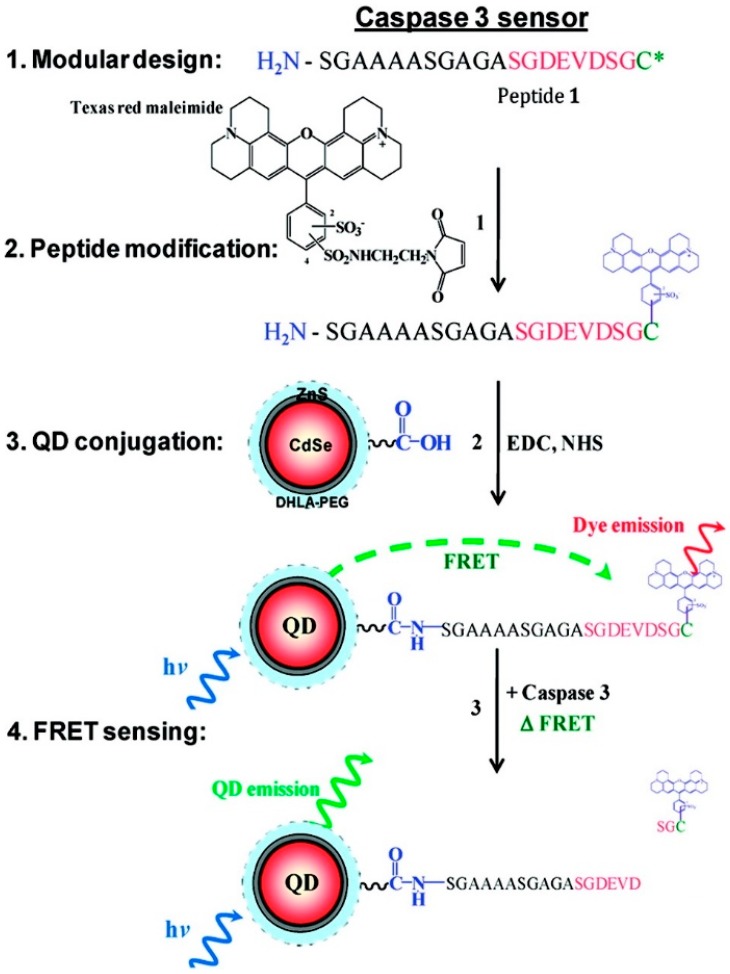
Schematic of the caspase 3 sensor design and response mechanism (Reprinted with permission from [76], Copyright 2010 American Chemical Society).

Caspase 3 is a member of the cysteine-aspartic acid protease family that plays an important role in programmed cell death (apoptosis) by cleaving a variety of key cellular proteins [72]. Caspase 3 activity has been proven to be involved in a variety of cancers such as breast, ovarian, and prostate, as well as Alzheimer’s disease [73,74,75]. Thus, developing biosensors for monitoring the activity of this enzyme *in vitro* and *in vivo* has attracted significant attention recently. Medintz and coworkers demonstrated peptide-based QD FRET sensors for monitoring proteolytic activity of caspase 3 [67,76]. In one of the designs, mCherry red fluorescent protein comprising an intervening N-terminal caspase 3 cleavage site was self-assembled on the surface of the QDs via the terminal His_6_-sequence of the peptide linker. Due to the close proximity of the fluorescent protein to the QDs, there is an efficient transfer of energy between them, but upon addition of the caspase 3 enzyme, the linker is cleaved and the fluorescent protein diffuses away causing a reduced FRET efficiency and a quantitative ratiometric change in donor-acceptor emissions [67]. In another design a peptide linker was engineered to have C-terminal cysteine at one end, an intervening DEVD sequence that serves as the caspase 3 cleavage site, and an N-terminal primary amine at the other end (Figure 3). The cysteine thiol group was used to label the peptide with Texas Red maleimide, and the amine end was used for conjugation of the dye-labeled peptide to the carboxyl groups present on the surface of the polyethylene glycol modified dihydrolipoic acid (DHLA-PEG) capped QDs using EDC. The response mechanism is again based on linker cleavage in the presence of caspase 3 and the change in the FRET efficiency that is proportional to the amount of the enzyme present [76]. 

**Figure 4:**
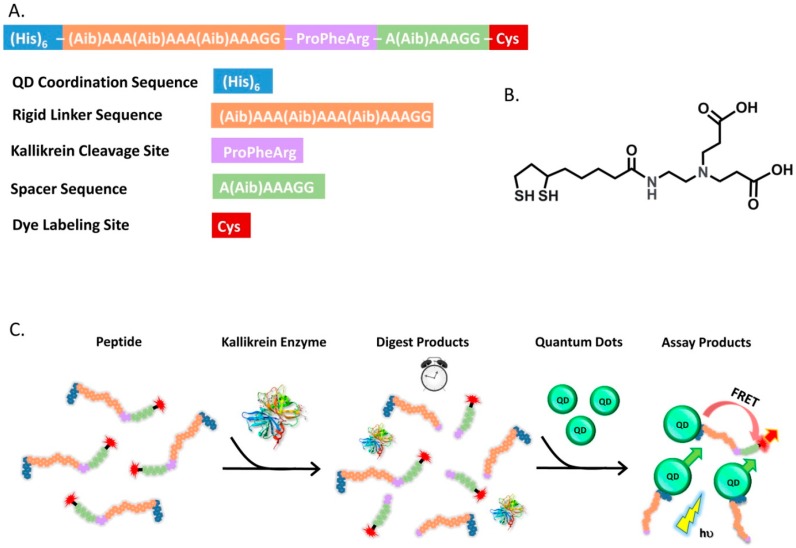
(**A**) Peptide sequence and sensor functional elements; (**B**) Chemical structure of the ligands on the QD surface; (**C**) Schematic illustration of the two-step assay for kallikrein proteolytic activity (Reprinted with permission from [77], Copyright 2014 American Chemical Society).

Medintz and coworkers recently reported a two-step assay for kallikrein proteolytic activity based on QD-FRET [77]. Kallikrein is a serine protease capable of initiating the blood clotting cascade. Aberrant activity of this enzyme in human plasma can potentially result from the presence of oversulfated chondroitin sulfate (OSCS) contamination, which is a drug adulterant in heparin. The design of the QD-based FRET sensor for kallikrein is challenging due to the fact that the large size of this enzyme causes a steric hindrance that prevents proper access and cleavage of the substrate near the QD surface. To overcome this challenge, the authors developed a two-step assay. The peptide substrate is composed of a terminal histidine tag that allows the self-assembly of the substrate on the QD surface, a kallikrein cleavage sequence, followed by a rigid spacer sequence with a N-terminal cysteine that provides a thiol group for labeling with a cyanine dye (Cy3). In the two-step assay shown in Figure 4, a known concentration of the Cy3-labeled kallikrein substrate peptide was digested in the presence of the enzyme, and then in second step, the sample was diluted with a buffer containing the QDs. The observed changes in QD-dye emission ratio due to FRET can be correlated to the concentration of the cleaved peptides over time.

### 3.3. Ratiometric Sensors, Structural Rearrangement 

**Figure 5:**
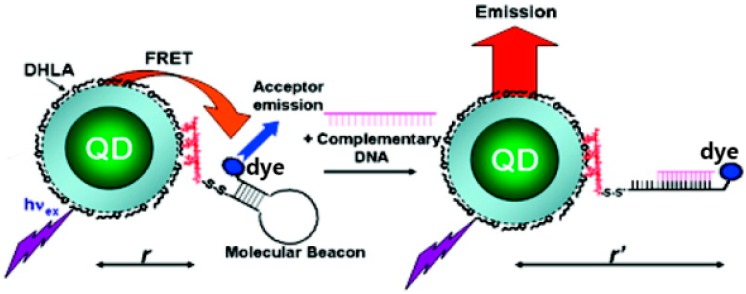
FRET is efficient between a nanocrystal and dye connected through a DNA hairpin linker. The addition of the complementary DNA (the analyte) changes the distance between the QD-dye complex. (Reprinted with permission from [84], Copyright 2007 American Chemical Society).

Another ratiometric sensing strategy is based on analyte-induced structural changes in a linker that connects a QD-dye FRET pair (Figure 5). Such a design is ideal for the use of a DNA molecular beacon due to its hairpin structure [78,79,80,81,82,83,84]. The dye-labeled end is initially in close proximity to the QD. Upon addition of the target DNA and hairpin unfurling the dye-labeled end moves away from the QD resulting in a FRET efficiency decrease and subsequent QD PL emission recovery (Figure 5).

Using DNA to link QDs and FRET accepting dyes can perform more functions other than to detect the presence of complementary DNA. Recently, Kay *et al.* reported on the usage of a cytosine-rich oligonucleotide to bridge QDs and a rhodamine dye [85]. The DNA fragment was chosen because it is known to undergo conformational changes as a result of pH which results in a greater QD-dye distance under basic conditions. The group also successfully used this system to measure the acidification of maturing HeLa cell endosomes.

## 4. The Response Mechanisms of QD-Based FRET Sensors: Spectral Overlap

Another QD-FRET sensor design involves the changes in the acceptor dye photophysical properties in response to the presence of the target analyte. For example, since FRET depends on spectral overlap of the QD emission with the dye absorption spectrum, any changes in absorption spectrum of the dye such as cross sectional or wavelength shifts can alter the FRET efficiency. The change in the dye optical properties may result from a chemical reaction with the analyte or perhaps by an alteration of the dye’s microenvironment. This in turn alters the ratio of the QD to dye emission that can be correlated to the analyte concentration. This strategy is very facile for the detection of small chemical elements such as hydrated salts or pH levels [76,86,87]. 

### 4.1. pH

**Figure 6:**
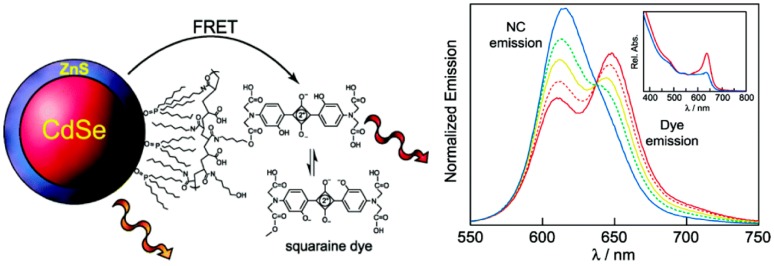
Schematic illustration of the sensor construction and the pH-dependent emission profiles. The squaraine absorption suppression in basic condition is shown in the inset (Reprinted with permission from [88], Copyright 2006 American Chemical Society).

The first ratiometric QD-based FRET pH sensor was reported by Snee *et al.* [88]. The system was constructed using CdSe/ZnS QDs encapsulated with a hydrophobically modified poly(acrylic acid) that is conjugated to squaraine dye via an ester bond (Figure 6). FRET is established between the coupled chromophores as verified by time resolved measurements that found the luminescence decay timescales of the blank QD *versus* QD-dye shortened from 31 ns to 19 ns. The absorption spectrum of the squaraine is pH-dependent because of the (de)protonation of phenolic groups around the squaraine functionality. At high pHs the FRET efficiency decreases due to a lowering of the dye absorption resulting in an emission spectrum that is dominated by QD luminescence. At low pHs the opposite occurs, resulting in quenching of the QD emission due to more energy transfer to the dye. The ratiometric response of the sensor offers advantages over single intensity based response due to less sensitivity to light fluctuations and the fact that dual-emissive ratiometric probes are self-calibrating. The sensor was also used to measure pH in a highly scattering medium, with results that were within a 5% margin of error.

**Figure 7:**
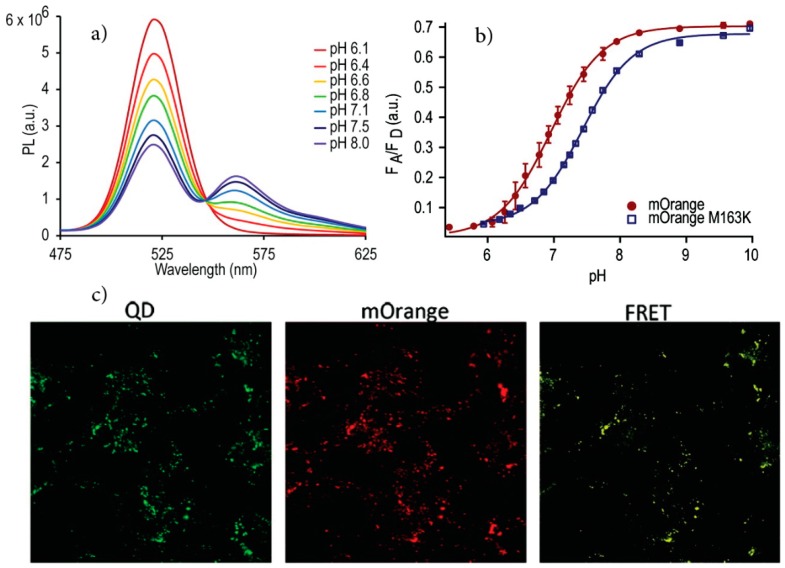
(**a**) QD-mOrange FP probe emission modulation as a function of pH; (**b**) pH-dependent integrated acceptor to donor emission ratio; (**c**) Fluorescence microscopy images after delivery of the sensor using filter sets to image the QDs separately from the pH sensitive FP. (Reprinted with permission from [86], Copyright 2012 American Chemical Society).

Several systems for QD-based ratiometric sensing using coupled QD-sensing dye chromophores have since been reported that follow this strategy. Dennis *et al.* [86] used pH-sensitive fluorescent proteins (FPs) for the fabrication of QD-FP FRET based pH sensors. In this study mOrange and its homologue mOrange M163K, which is a mutant with a shifted pKa and improved photostability, were conjugated to carboxyl-functionalized QDs using carbodiimide chemistry. The spectral overlap between the QD emission and FP absorption resulted in energy transfer from the QD to the proteins that are sensitive to pH changes. This is a result of the pH dependence of the molar absorptivity of these proteins. Increasing the solution pH caused an increase in the acceptor to donor emission ratio which was shown to be due to modulation of the FRET efficiency (Figure 7). The sensors are functional over the pH range of 6–8 and display the maximum sensitivity near the pKa of the acceptor fluorescent proteins. Indirect excitation of the FPs by FRET also increased their photostability and made simultaneous cell imaging/pH sensing possible. To measure the intracellular pH, the QD-FP conjugates were further modified with polyarginine to promote endosomal uptake. After incubation with HeLa cells and washing, the emission of the endosomal pH sensors was monitored over time using filters to image the QD emission separately from the dye emission. The results indicated that the pH drops as the early endosome matures to a late endosome as expected. Furthermore, all controls confirmed that the results were not due to photobleaching or degradation of the proteins rather than the natural pH change (acidification) that endosomes undergo.

Krooswyk *et al.* [89] studied the response mechanism of this QD-sensing dye motif using a CdS/ZnS-fluorescein pH sensor. They initially proposed that the response mechanism should be dictated only by organic dye’s photochemical properties. And while the pH response of the neat dye was consistent with the Henderson-Hasselbalch equation, as it should, the QD-dye conjugate pH-dependent fluorescence emission ratio deviated from the typical sigmoidal shape. As a result, it was found that the conjugation of the organic dye to a QD significantly changes the dye’s microenvironment. Taking into account the variables that can affect the FRET efficiency and subsequent response of the QD-dye pH sensor, it was determined that the response is a function of the QD size as this changes the nanocrystal emission which in turn alters the FRET overlap integral, the swelling of the polymer used for QD encapsulation that changes the QD-to-dye distance [90], and the electrostatically different microenvironment that dye senses after conjugation into the QD. As such, it is very difficult to predict the performance of a QD-dye FRET sensor based on photochemical properties of the organic dye alone. 

### 4.2. Metals

**Figure 8:**
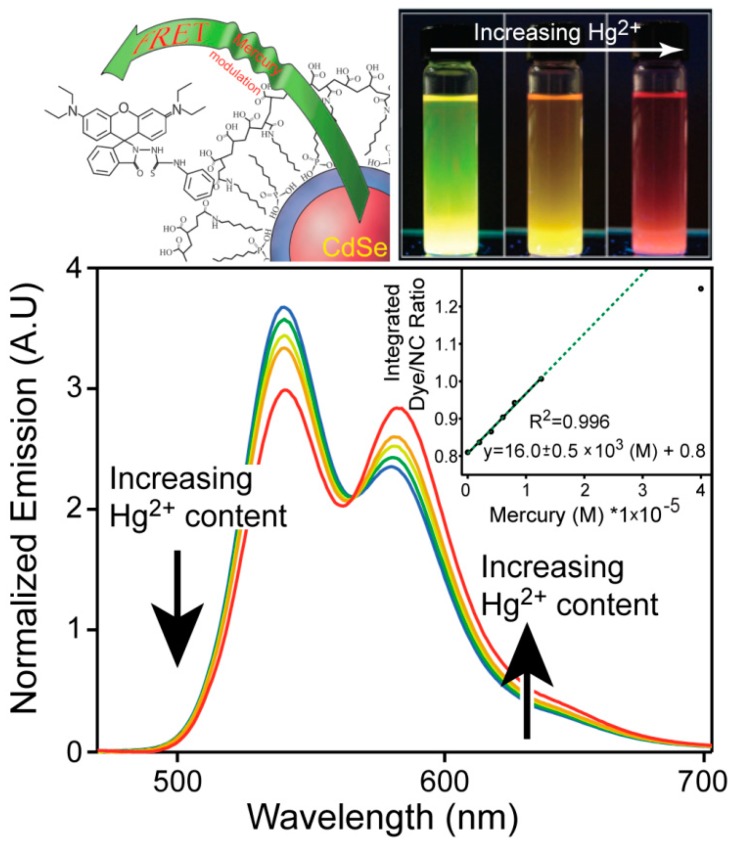
The ratiometric response of the sensor as a function of Hg^2+^ content (reprinted with permission from [98], Copyright 2011 The Royal Society of Chemistry).

Environmental contamination by metals is hazardous as some metal ions are extremely toxic. As such, the use of QDs for toxic metal sensing is topical, and many groups have reported on their usage. It has been well known that even the most well passivated aqueous QDs are quenched by cations such as Ag^+^ [91], Pb^2+^ [92], Fe^2+^ [93], Cu^2+^ [94,95] and Hg^2+^ [96] due to cation exchange [97]. As a result, quenching of QD fluorescence by these metals is highly non-specific and is not a practical analytical method in general. Furthermore, this non-selective ion quenching is an impediment to the development of a FRET sensor with QD donors due to direct and irreversible destruction of the donors by the analytes. Despite this, Page *et al.* [98] reported a ratiometric mercury sensor based on FRET between green emitting CdSe/ZnS QDs and a thiosemicarbazide functionalized rhodamine B dye (Figure 8). The dye has a unique role in that it reacts with mercuric ions to form mercury sulfide, which is highly insoluble in water. This prevents QD quenching by the metal. Furthermore, in the absence of Hg^2+^ ions the dye is optically silent due to disruption of the delocalized electronic structure of the dye by the thiosemicarbazide group. Thus, the QD is the dominant emitter in the QD-dye coupled chromophore. Upon exposure to Hg^+2^ ions the desulfurization reaction that creates HgS also results in the restoration of the dye’s absorption and emission properties, causing it to act as a good energy acceptor for the QD donor. Initially, an amine functional thiosemicarbaziderhodamine dye was developed for conjugation purposes, but the dye was very unstable and self-activated without mercuric ions. However, it was found that the thiosemicarbaziderhodamine dye had a very strong non-specific interaction with the surface of the polymer-coated QDs, so chemical coupling was unnecessary. It is worth noting that this QD protection mechanism is not perfect and QD emission quenching occurs via exposure to Hg^2+^ ions, which likely explains the high limit of detection (79 ppb). However, the motif of “sense and sequester” does function for its intended purpose.

Li *et al.* [99] reported a FRET system for detection of Hg^2+^ using aqueous CdTe QDs as donors and butylrhodamine B (BRB) as an acceptor. Since the electrostatic interaction between the electronegative QDs and cationic BRB dye was not strong enough for self-assembly to occur, QD-dye adsorption was promoted using surfactants. Specifically cetyltrimethylamonium bromide (CTMAB) formed micelles that enhanced the QD-dye interactions and improved the FRET efficiency. Energy transfer was confirmed by the significant enhancement of the BRB PL emission and corresponding quenching of the CdTe PL emission. Upon addition of Hg^2+^ to the FRET system, both the donor and the acceptor were quenched, but BRB was quenched more than the CdTe QDs. The response mechanism can be explained by cation exchange of Cd(II) by Hg(II) on the QD surface that results in fluorescence quenching of the CdTe and concomitant quenching of BRB PL emission due to the loss of FRET. The sensor showed linear response over a wide Hg^2+^ concentration range and had a detection limit of 20.3 nmol·L^−1^ (4.07 ppb). Another method to mitigate the quenching of QD donors for mercuric ion sensing is based on silica coatings to protect the nanocrystals. For example, Liu *et al.* [87] adapted this strategy by first embedding CdTe QDs inside silica particles using a microemulsion method. The nanocrystals can still be quenched by Hg^2+^, so the particles were then given a thin cationic shell that electrostatically prevented mercury absorption. The surface was also linked to a spirolactamrhodamine B derivative that served as the Hg^2+^ probe. The response mechanism is based on ring-opening of the spirolactam due to complexation with Hg^2+^, which results in FRET between the QD and activated dye that provided a ratiometric response. Time resolved emission also was used to demonstrate energy transfer from the QDs to the activated dye. The only negative aspect of this work is that the necessary cationic shell was detrimental to the detection limit that was found to be 260 nmol·L^−1^ (52 ppb). There are other examples of using silica embedded QDs to ratiometrically detect mercuric ions [100,101], however these are not FRET based systems.

### 4.3. Proteins and Enzymes

**Figure 9:**
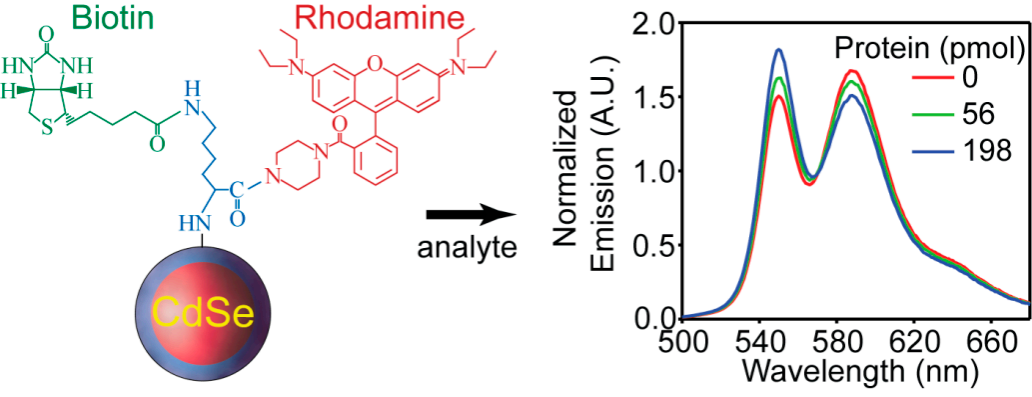
Schematic illustration of the protein sensor structure composed of the CdSe/ZnS QD conjugated with RB piperazine-lysine-biotin, and the ratiometric response of the sensor to streptavidin (reprinted with permission from [105], Copyright 2014 The American Chemical Society).

The enzyme-linked immunosorbent assay (ELISA) [102] is ubiquitous for the detection of proteins that are biomarkers for a variety of diseases such as influenza, cancer, and HIV. The methodology is heterogeneous and generally relies on multiple antibody-antigen interactions. Specifically, a substrate-bound capture antibody is used to immobilize its antigen whereupon another species-specific antibody-enzyme conjugate is used to catalyze the formation of an absorptive or fluorescent indicator. Highly engineered ELISA assays are very sensitive towards their targets, but the use of secondary antibodies is somewhat time-consuming and difficult to perform due to the multiple washing and blocking steps involved. As such, the development of QD protein sensors that function outside of the ELISA paradigm are if interest [56,80,103,104]. Recently Tyrakowski *et al.* demonstrated a novel paradigm of ratiometric sensing of proteins using QD FRET-based sensors without a secondary antibody requirement [105]. The system is composed of a three spoke wheel-like structure where rhodamine B piperazine, biotin (a streptavidin agonist), and a QD are all conjugated together with lysine (Figure 9). The QD and dye are in close proximity and both chromophores are emissive due to energy transfer. Binding of the protein streptavidin to biotin brings the protein in close proximity to the dye. This in turn alters the microenvironment of the dye and causes quenching that results in a ratiometric response (Figure 9). The sensor responds specifically to streptavidin, and other controls such as a similar system without biotin show no response towards the protein. The detection limit is calculated to be in the same range of ELISA methods, and it can be lowered by diluting the sensor concentration which is a known benefit of ratiometric sensors. This detection method is fast and easy to perform due to the homogeneity of the sensing platform. The authors also showed that the method can be adapted to detect other proteins using DNA oligonucleotide aptamers. They reported a similar sensing mechanism for the detection of thrombin using thrombin binding aptamer coupled to TAMRA as the energy accepting dye. At the same time Zhang *et al.* reported a nearly-identical system for thrombin sensing where protein binding displaced a FRET-accepting dye bound to the aptamer [106].

### 4.4. Oxygen

**Figure 10:**
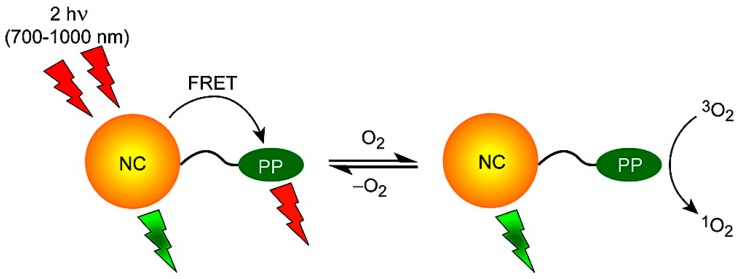
Schematic illustration of a two-photon absorbing QD-FRET oxygen sensor (reprinted with permission from [120], Copyright 2009 The American Chemical Society).

Tumors develop in a state of hypoxia due to rapid growth, and oxygen levels inside the tumor can be indicators of tumor health and the effectiveness of treatment. Therefore the development of reliable oxygen sensors to probe biological environments has attracted significant attention. There are many reports on oxygen sensitive materials, generally composed of heavy metal complexes [107,108], metaloporphyrins [109,110], and polycyclic aromatic hydrocarbons [111,112]. All of these materials have room temperature phosphorescence emission that is quenched by the presence of oxygen and generate singlet oxygen in the process [113], which is known to have a destructive effect on cancer tumor cells and can also be used as photodynamic therapy [114,115]. There are many reports on the successful conjugation of photosensitizers such as rose bengal, chlorin e6, and phthalocyanine with QDs that serve as FRET donors to generate singlet oxygen for therapeutic purposes [115,116,117,118,119]. Considering the unique electro-optical properties of QDs, they can serve as both a versatile platform for immobilizing oxygen sensitive materials and as a light absorption and excitation source of the phosphors. QDs exhibit higher absorption cross-sections compared to phosphors, and thus FRET-driven excitation of the phosphors can have a higher yield compared to direct excitation. As a well-passivated nanocrystal material is not intrinsically oxygen sensitive, the QD acts as more of an internal reference however this still makes a ratiometric response possible. McLaurin *et al.* [120] reported a QD-FRET ratiometric oxygen sensor designed to detect oxygen in biological environments. Two osmium (II) polypyridyl complexes with amine functional linkers were synthesized as the oxygen sensitive materials. They were then conjugated to the QD via amide bond formation. The QDs were designed to function as FRET donors by engineering an overlap with the osmium (II) complexes’ broad absorption spectra and the narrow QD narrow emission. Excitation was also performed using two-photon absorption, which allows for deeper tissue penetration and more selectivity in terms of localizing the excitation (Figure 10).

Lemon *et al.* [121] reported a similar QD-FRET oxygen sensor using palladium porphyrins as oxygen sensitive materials. The QD-porphyrin conjugates were constructed by self-assembly of Pd porphyrins on the QD surface through meso-pyridyl substituents. Excitation of the Pd porphyrins is via FRET and the ratiometric response to the presence of oxygen is based on the proportional quenching of the Pd porphyrin emission. Ingram *et al.* [122] reported the construction of a ratiometric optode for oxygen detection based on FRET from a QD to platinum (II) octaethylporphine ketone. A combination of polyvinyl chloride and bis(2-ethylhexyl)sebacate was used as a biologically inert and O_2_ permeable polymer matrix for fabrication of the optode. The optode was then used for the real-time extracellular measurement of O_2_ in biological microdomains of active brain tissue. 

## 5. The Response Mechanisms of QD-Based FRET Sensors: High Dye Loading Levels

**Figure 11:**
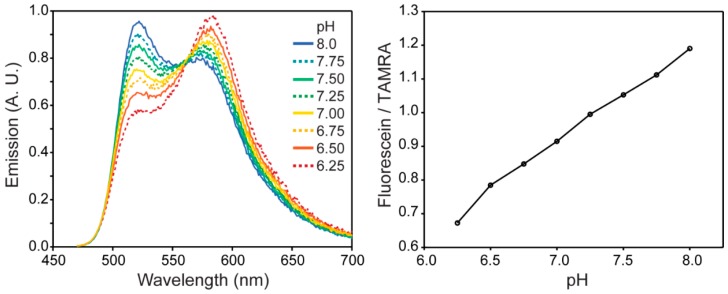
Coupling fluorescein and TAMRA dyes to the surfaces of Fe_2_O_3_ nanoparticles creates a FRET pair between the two chromophores to produce a ratiometric emission spectrum as a function of pH.

There is a significant level of interest in the synthesis of nanomaterials that are simultaneously fluorescent and magnetic. Unfortunately, some basic properties of semiconductor photophysics generally dictate that magnetic materials have fast de-excitation pathways that prevent fluorescence. Hence, there is a bifurcation in research where magnetic semiconductor nanomaterials are generally only used for MRI contrast agents whereas fluorescent quantum dots are used for visible light imaging. Our group recently addressed this issue by coating Fe_2_O_3_ nanocrystals [123] with fluorescein dye to make a magnetic nanomaterial that functioned as a pH sensor [18]. Recently we added another dimension to this where fluorescein and amine-functional carboxytetramethylrhodamine (TAMRA) were both conjugated to the surface of magnetic iron oxide nanomaterials. As shown in Figure 11, the system responds ratiometrically to a change in pH which is fully calibratable. There is a FRET interaction between the dye coupled chromophores as evident from the PLE spectrum of the TAMRA dye which showed fluorescein-like features. Furthermore, we can attach hundreds of dyes per dot [30], which mitigates photobleaching of the organic chromophores. 

## 6. Conclusions and Outlook

Semiconductor quantum dots offer advantages for use in analytical applications as FRET-based chemosensors and biosensors. Due to their unique electro-optical properties and recent developments on numerous water-solubilization and conjugation strategies, QDs provides a versatile platform for FRET-based sensing probes. Current studies have shown that QDs can be effectively employed as FRET donors to organic dyes and fluorescent proteins, offering advantages such as narrow symmetric emission with minimum spectral bleed-through and broad absorption spectra. This allows for excitation of the QDs donor without direct excitation of the acceptor(s). In some special cases QDs can also play the acceptor role with some lanthanides which is useful in time-resolved fluoro-immunoassays. Despite of the remarkable developments towards the use of the QDs in biosensing, there is still a demand for a simple and practical strategy for the intracellular delivery of these QD-based biosensors [124]. Another direction that the community is moving towards is cadmium free nanocrystals; however, it should be noted that the toxicology of cadmium materials does not appear to be an issue even in animal studies [125].
